# Exposome and unhealthy aging: environmental drivers from air pollution to occupational exposures

**DOI:** 10.1007/s11357-023-00913-3

**Published:** 2023-09-09

**Authors:** Tamas Pandics, David Major, Vince Fazekas-Pongor, Zsofia Szarvas, Anna Peterfi, Peter Mukli, Rafal Gulej, Anna Ungvari, Monika Fekete, Anna Tompa, Stefano Tarantini, Andriy Yabluchanskiy, Shannon Conley, Anna Csiszar, Adam G. Tabak, Zoltan Benyo, Roza Adany, Zoltan Ungvari

**Affiliations:** 1 Department of Public Health, Faculty of Medicine, Semmelweis University, Budapest, Hungary; 2 Department of Public Health Laboratory, National Public Health Centre, Budapest, Hungary; 3 Department of Public Health Siences, Faculty of Health Sciences, Semmelweis University, Budapest, Hungary; 4 Vascular Cognitive Impairment, Neurodegeneration and Healthy Brain Aging Program, Department of Neurosurgery, University of Oklahoma Health Sciences Center, Oklahoma City, OK USA; 5 Oklahoma Center for Geroscience and Healthy Brain Aging, University of Oklahoma Health Sciences Center, Oklahoma City, OK USA; 6 Stephenson Cancer Center, University of Oklahoma, Oklahoma City, OK USA; 7 Department of Health Promotion Sciences, College of Public Health, University of Oklahoma Health Sciences Center, Oklahoma City, OK USA; 8 International Training Program in Geroscience, Doctoral School of Basic and Translational Medicine/Department of Public Health, Semmelweis University, Budapest, Hungary; 9 Department of Cell Biology, University of Oklahoma Health Sciences Center, Oklahoma City, OK USA; 10 UCL Brain Sciences, University College London, London, UK; 11 Department of Internal Medicine and Oncology, Faculty of Medicine, Semmelweis University, Budapest, Hungary; 12 Department of Translational Medicine, Semmelweis University, Budapest, Hungary; 13 Eötvös Loránd Research Network and Semmelweis University (ELKH-SE) Cerebrovascular and Neurocognitive Disorders Research Group, Budapest, H-1052 Hungary; 14 ELKH-DE Public Health Research Group, Department of Public Health and Epidemiology, Faculty of Medicine, University of Debrecen, 4032 Debrecen, Hungary; 15 Epidemiology and Surveillance Centre, Semmelweis University, 1085 Budapest, Hungary

**Keywords:** Exposome, Aging, Environmental pollution, Toxicology, Accelerated aging, Biological age

## Abstract

The aging population worldwide is facing a significant increase in age-related non-communicable diseases, including cardiovascular and brain pathologies. This comprehensive review paper delves into the impact of the exposome, which encompasses the totality of environmental exposures, on unhealthy aging. It explores how environmental factors contribute to the acceleration of aging processes, increase biological age, and facilitate the development and progression of a wide range of age-associated diseases. The impact of environmental factors on cognitive health and the development of chronic age-related diseases affecting the cardiovascular system and central nervous system is discussed, with a specific focus on Alzheimer’s disease, Parkinson’s disease, stroke, small vessel disease, and vascular cognitive impairment (VCI). Aging is a major risk factor for these diseases. Their pathogenesis involves cellular and molecular mechanisms of aging such as increased oxidative stress, impaired mitochondrial function, DNA damage, and inflammation and is influenced by environmental factors. Environmental toxicants, including ambient particulate matter, pesticides, heavy metals, and organic solvents, have been identified as significant contributors to cardiovascular and brain aging disorders. These toxicants can inflict both macro- and microvascular damage and many of them can also cross the blood–brain barrier, inducing neurotoxic effects, neuroinflammation, and neuronal dysfunction. In conclusion, environmental factors play a critical role in modulating cardiovascular and brain aging. A deeper understanding of how environmental toxicants exacerbate aging processes and contribute to the pathogenesis of neurodegenerative diseases, VCI, and dementia is crucial for the development of preventive strategies and interventions to promote cardiovascular, cerebrovascular, and brain health. By mitigating exposure to harmful environmental factors and promoting healthy aging, we can strive to reduce the burden of age-related cardiovascular and brain pathologies in the aging population.

## Introduction

The global population is experiencing a significant increase in the number and proportion of individuals aged 60 years and older. According to the World Health Organization (WHO), the number of people over 60 years old reached 1 billion in 2019 and is projected to surpass 2.1 billion by 2050 [1]. This demographic shift is accompanied by a rising prevalence of age-related non-communicable diseases (NCDs) in aging societies, particularly in the Western world. These NCDs include cardiovascular and cerebrovascular diseases (such as heart failure, myocardial infarction, stroke, vascular cognitive impairment), cancers, chronic respiratory diseases (like chronic obstructive pulmonary disease), chronic kidney disease, type-2 diabetes mellitus, musculoskeletal diseases, and neurodegenerative diseases. The impact of these chronic age-related NCDs on the quality of life for affected individuals spans several decades and carries substantial socioeconomic implications for Western societies. Deterioration of cognitive health associated with age-related NCDs is especially important in that regard.

In the USA, over 90% of older individuals have at least one chronic NCD, with approximately three-quarters experiencing two or more [2]. The economic burden associated with age-related chronic diseases has been estimated at a staggering $47 trillion for the period from 2010 to 2030 [3]. The financial strain imposed by the costly care required for older individuals with age-related NCDs affects pension systems and healthcare systems alike. Recognizing the magnitude of this problem, the World Health Organization emphasizes the importance of focusing on prevention of age-related NCDs and the promotion of healthy aging [4, 5].

**Fig. 1:**
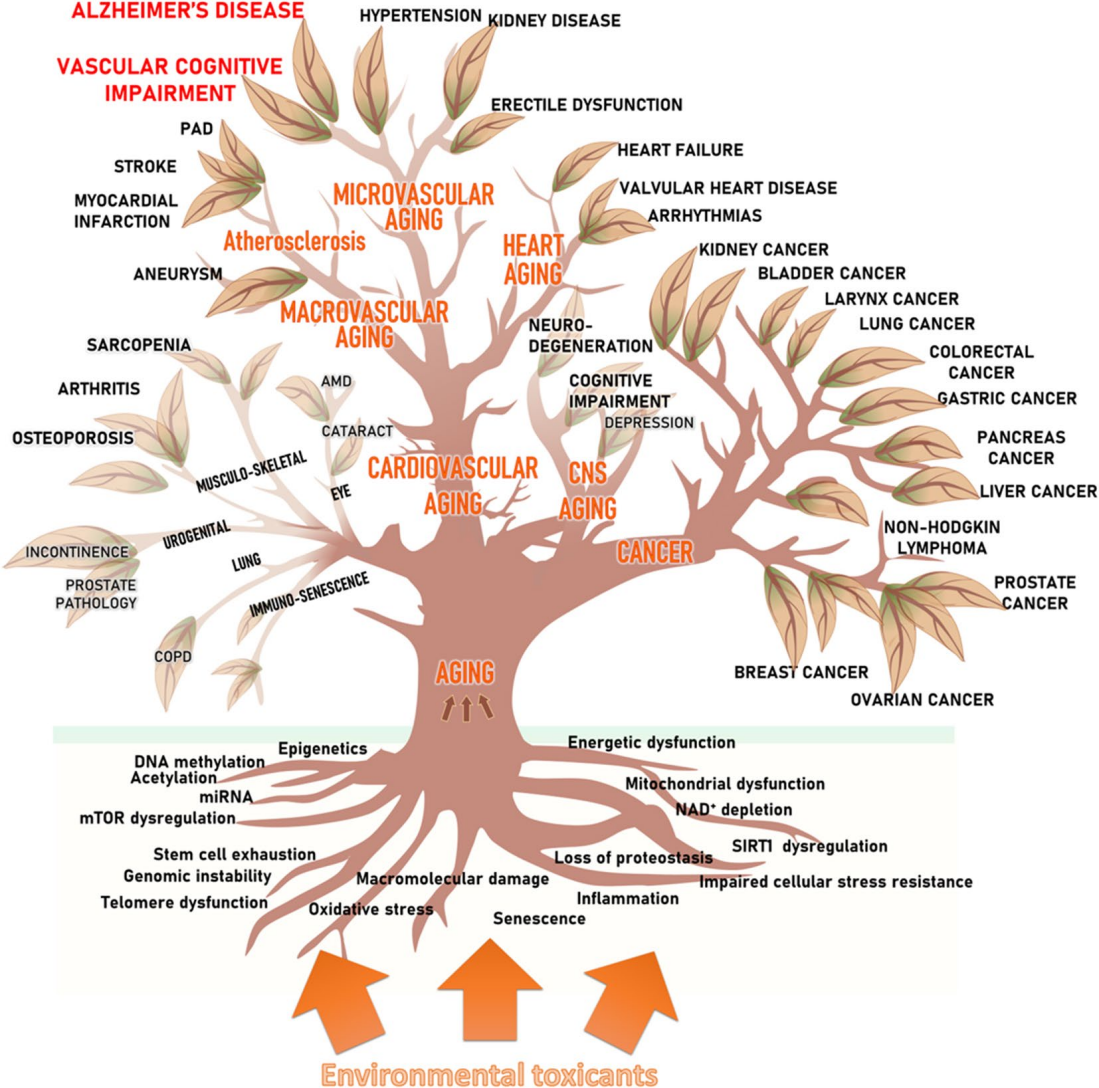
Conceptual model illustrating the contribution of environmental drivers to unhealthy aging, characterized by accelerated aging processes, increased biological age, and the development and progression of various age-associated diseases. Environmental toxicants (depicted in orange) play a key role in promoting age-related cardiovascular, cerebrovascular, and brain pathologies, as well as the pathogenesis of age-associated diseases in other organ systems. These toxicants exacerbate fundamental molecular and cellular aging processes (depicted as roots), which serve as the underlying mechanisms. The consequences of accelerated vascular aging induced by toxicants give rise to the genesis of micro- and macrovascular pathologies, including atherosclerotic vascular diseases, cerebral small vessel disease, and Alzheimer’s disease. Furthermore, many of these toxicants have the ability to cross the blood–brain barrier, leading to neurotoxic effects, neuroinflammation, and neuronal dysfunction, promoting the genesis of neurodegenerative diseases and cognitive decline. Clinical disciplines, biogerontology, and environmental toxicology, along with public health research, traditionally focus on specific age-related diseases (depicted as leaves), mechanisms of aging (depicted as roots), and environmental risk factors, respectively. Geroscience, as an integrative scientific field, considers the interaction between all these levels, facilitating a comprehensive understanding of the complex relationship between environmental factors and unhealthy aging

In recent years, advances in geroscience have led to a paradigm shift in our understanding of the pathogenesis of chronic age-related NCDs [6, 7]. It is now recognized that all age-associated diseases share common underlying cellular and molecular mechanisms of aging. These mechanisms include increased oxidative stress, cellular mitochondrial and energetic dysfunction, impaired cellular stress resilience, genetic instability and DNA damage, induction of cell senescence, heightened state of inflammation, epigenetic dysregulation, altered proteostasis, disruption of intercellular communication (including endocrine changes), stem cell dysfunction, and dysregulation of energy sensing pathways [6, 8–10] (Fig. 1). These pathways are genetically determined, but environmental and lifestyle factors play a critical role in modulating the rate of cellular and organismal aging, thereby defining the trajectories of age-related functional decline (Fig. 1). It is now emerging that environmental and lifestyle risk factors can exacerbate fundamental cellular and molecular aging processes, promoting accelerated aging phenotypes and the premature development of chronic age-related NCDs (Fig. 1).

The role of the environment in controlling human aging is increasingly being explored within an exposomic framework [11]. The exposome, as originally proposed by Dr. Wild in 2005 [12], encompasses all environmental exposures from conception that influence health outcomes. It encompasses internal factors (e.g., physical activity, microbiome, metabolism), general external factors (e.g., education, social status, climate, urban–rural environment), and specific external factors (environmental pollutants and chemical contaminants, lifestyle factors, occupation). It is worth noting that in our developed world, the majority of the population is exposed to approximately 80,000 to 100,000 artificially produced substances in their daily lives. In heavily polluted work environments, this number can reach up to 200,000 substances. The estimated number of “manmade” xenobiotic compounds currently stands at 10 million, highlighting the extensive exposure to synthetic chemicals in our modern society. These environmental toxicants have the potential to disrupt physiological processes and promote the pathogenesis of NCDs. Understanding the impact of this vast array of chemical exposures on aging and age-related diseases is critical for public health. Studies employing environmental epidemiology approaches, including geographical data linkages, have confirmed the significant contribution of exposure to environmental pollutants, including particulate matter in air pollution and occupational exposures, to the shared exposome, influencing longevity, geographic clustering, and the development of various age-related chronic NCDs such as neurodegenerative diseases, cardiovascular diseases, and cancer [11, 13–23].

Notably, exposure to environmental toxicants has been shown to associate with molecular hallmarks of aging [24–31]. In this review, we delve into the pathophysiological roles of environmental toxicants in modulating the fundamental cellular and molecular mechanisms of aging. Specifically, we examine their contributions to the pathogenesis of age-related NCDs, with a particular focus on cardiovascular, cerebrovascular and brain pathologies (Fig. 1). The selection of exposure factors considered in this review is based on an extensive literature search, encompassing a wide range of environmental toxicants, including ambient particulate matter, pesticides, heavy metals, and organic solvents, which have been previously identified as significant contributors to aging disorders in both the cardiovascular system and the brain. By exploring the interconnectedness between potential mechanisms of cardiovascular, cerebrovascular, and brain aging and the pathways affected by environmental factors, we gain insights into the complex relationships between environmental toxicants and unhealthy aging processes. Additionally, we explore the interaction between these cellular and molecular aging processes and disease-specific pathways. By examining the intricate relationships between environmental toxicants and cardiovascular, cerebrovascular, and brain aging, we can identify potential targets for public health interventions aimed at promoting healthy aging. By implementing preventive measures and interventions to mitigate the detrimental effects of environmental toxicants, we can strive to improve the overall well-being of the aging population. Our comprehensive review aims to provide valuable insights into the multifaceted role of environmental drivers in aging and guide future research efforts and public health strategies for healthy aging.

## Exacerbation of chronic age-related diseases by environmental toxicants

From a geroscience perspective, certain “risk factors” contribute to the pathogenesis of NCDs by exacerbating cellular and molecular mechanisms of aging (Fig. 1). Among these factors, environmental toxicants have been found to have significant detrimental effects on biological aging processes [20, 23, 24, 26–35]. Research indicates that environmental toxicants play a critical role in the development of NCDs, with the World Health Organization (WHO) estimating that 24% (13.7 million) of all global deaths per year are linked to environmental factors [36]. Within this number, 8.5 million deaths are attributed to NCDs, and the top three causes related to the environment are ischemic heart disease, chronic respiratory diseases, and cancer. In the following sections, we will briefly discuss the interaction between environmental toxicants, aging processes, and the genesis of organ-specific NCDs.

### Cardiovascular diseases

Vascular aging contributes to the age-dependent rise in a broad range of macrovascular and microvascular pathologies, including hypertension, atherosclerotic diseases (such as ischemic heart disease, peripheral artery disease, and stroke), aortic aneurysms, heart failure, cerebral small vessel disease, and age-related microvascular pathologies affecting other organs (such as glomerulosclerosis, microvascular rarefaction, and retinal pathologies) [8, 37, 38]. Aging alone confers a significantly higher risk for these diseases compared to “conventional” risk factors like lipid levels, smoking, diabetes mellitus, and sedentary lifestyle [8, 37].

The cellular and molecular mechanisms of aging that contribute to the pathogenesis of age-related cardiovascular diseases have been the subject of recent reviews [8, 37, 39]. Increased oxidative stress plays a significant role in vascular aging [8, 37, 40–51]. The elevated production of reactive oxygen species (ROS) leads to endothelial dysfunction by inactivating endothelium-derived nitric oxide (NO) and producing peroxynitrite (ONOO-) [8, 37, 52]. Consequently, it results in age-related reduction in endothelium-dependent dilation [53, 54], enhanced vasoconstriction, and dysregulation of tissue perfusion [8, 37]. The lack of NO and increased oxidative stress also promotes vascular inflammation and the development of a proatherogenic vascular phenotype in aging [8, 37, 50]. Increased oxidative stress can activate matrix metalloproteinases, which disrupt the structural integrity of aged arteries, potentially contributing to large artery stiffening and the pathogenesis of aortic aneurysms and cerebral microhemorrhages [37, 44, 49, 55–59]. Oxidative stress is also associated with DNA damage and DNA damage–induced cellular senescence, which are additional mechanisms of aging contributing to vascular pathologies [37, 60, 61].

Mitochondrial alterations, including mitochondrial ROS production, impaired mitochondrial biogenesis, impaired cellular energy production, and mitochondrial DNA damage, further exacerbate vascular aging processes [8, 37, 39, 62–69]. Additionally, the release of several proinflammatory molecules increases with aging, leading to macrovascular and microvascular pathologies such as atherogenesis, aneurysm formation, and microvascular dysfunction [37].

The role of Nrf2, which coordinates an adaptive antioxidant response, has also been emphasized in recent studies [70–80]. Aging causes Nrf2 dysfunction in the vasculature, impairing the oxidative stress resilience of aged cells and contributing to age-related vascular pathologies induced by pro-oxidative, DNA damaging stimuli [75–77]. Aging also impacts the proteostasis system of the vasculature by impairing chaperones, the ubiquitin–proteasome system, and the lysosome-autophagy system [8, 37]. Furthermore, endothelial senescence contributes to endothelial dysfunction in aging and pathophysiological conditions associated with accelerated vascular aging [8, 37, 46, 73, 81–83]. Epigenetic alterations, such as changes in DNA methylation patterns or miRNA dysregulation, also contribute to impairment of angiogenic processes, vascular inflammation, or atherogenesis [8, 37].

The heart and vascular system are highly vulnerable to various environmental agents. The Global Burden of Diseases, Injuries, and Risk Factors Study (GBD) estimated that long-term exposure to ambient fine particulate matter (PM2.5) contributed to 2.9 million deaths globally each year, with nearly 50% of these deaths attributable to ischemic heart disease and stroke [84]. Even at concentrations below current regulatory standards in the USA and European Union, long-term exposure to PM10 and PM2.5 within metropolitan areas is associated with the progression of coronary calcification, indicating accelerated atherosclerosis [85]. Air pollution has been identified as a risk factor for ischemic heart disease [86] and stroke [87, 88]. Ambient air pollution and household air pollution from cooking with polluting fuels are estimated to cause 13 and 17% of cardiovascular diseases, respectively [89–91]. Acrolein, a highly reactive unsaturated aldehyde, is generated during the burning of diesel fuels, gasoline, woods, plastics, cigarette smoking, and frying of food with fats. The Environmental Protection Agency classifies acrolein as a high-priority air and water toxicant. Strong evidence suggests that acrolein can cause damage to cardiac myocytes and endothelial cells, promoting endothelial dysfunction, vascular disease, and heart failure [92–95].

Smoking and exposure to secondhand smoke play significant roles in the pathogenesis of cardiovascular diseases [96, 97]. Cigarette smoke contains numerous harmful substances, including carbon monoxide, nicotine, and a range of toxic chemicals and particulate matter. Smoking is a well-established risk factor for cardiovascular diseases, particularly ischemic heart disease, peripheral artery disease, stroke and vascular cognitive impairment [96, 97]. The detrimental effects of smoking on the cardiovascular system arise from multiple mechanisms [98–109]. Firstly, smoking promotes the development of atherosclerosis by damaging the endothelial lining of blood vessels, increasing inflammation, and accelerating the formation of fatty deposits within the arterial walls [96, 97]. Secondly, smoking leads to increased oxidative stress and endothelial dysfunction, impairing the production and bioavailability of NO and promoting nitrative stress [96, 97, 103, 110, 111]. Increased oxidative stress also contributes to lipid oxidation, induces expression of adhesion molecules of the endothelium and activates macrophages and platelets [96, 97, 110, 111]. Macrophages engulf oxidized lipids, leading to the formation of foam cells within the aortic wall. Subsequent foam cell death triggers the release of these lipids, promoting the development of lipid-rich aortic plaques [96]. Furthermore, cigarette smoke leads to an increased expression and activity of matrix metalloproteinases (MMPs), as well as to decreased expression of MMP inhibitors causing alterations of vascular extracellular matrix and tissue remodeling that play a significant role not just in atherogenesis but in the formation of aneurysms as well [96]. Moreover, smoking promotes platelet aggregation and blood clot formation, increasing the risk of thrombotic events. Additionally, smoking causes an unfavorable lipid profile, with lower levels of beneficial high-density lipoprotein (HDL) cholesterol and elevated levels of harmful low-density lipoprotein (LDL) cholesterol. Furthermore, smoking induces systemic inflammation and adversely affects the balance of various pro-inflammatory and anti-inflammatory molecules, contributing to the progression of cardiovascular diseases [98, 102, 103, 112–114]. Results from the Cardiovascular Health Study suggest that smoking-induced inflammation promotes cardiomyocyte injury exacerbating heart failure [115]. Secondhand smoke, which is the involuntary inhalation of smoke emitted by smokers, also poses a significant risk to cardiovascular health. Exposure to secondhand smoke has been associated with an increased risk of developing cardiovascular diseases, similar to active smoking. The toxic components in secondhand smoke can lead to endothelial dysfunction, inflammation, and increased oxidative stress in non-smokers, thereby contributing to the pathogenesis of cardiovascular diseases. Overall, the avoidance of smoking and secondhand smoke exposure is crucial for preventing and reducing the burden of cardiovascular diseases.

Water pollution is a significant environmental concern that can have adverse effects on cardiovascular health. Various pollutants and contaminants found in water sources can increase the risk of cardiovascular diseases [116]. For example, arsenic in drinking water has been identified as a potential risk factor for cardiovascular diseases [117]. Prolonged exposure to elevated levels of arsenic in drinking water has been associated with an increased incidence of hypertension, atherosclerosis, and cardiovascular mortality [117]. Similarly, exposure to lead and cadmium, often found in contaminated water sources, has been linked to an elevated risk of developing cardiovascular diseases [118–126]. Other pollutants present in water pollution that can pose harm to the cardiovascular system include mercury. Mercury is a toxic heavy metal that can contaminate water sources, primarily through industrial processes and the burning of fossil fuels. Chronic exposure to mercury has been associated with an increased risk of cardiovascular diseases, including hypertension, coronary artery disease, and myocardial infarction [127–134]. Water pollution may also contain various organic pollutants, such as polycyclic aromatic hydrocarbons (PAHs), pesticides, and industrial chemicals. These substances have been linked to adverse cardiovascular effects, including endothelial dysfunction, oxidative stress, inflammation, and disruption of cardiac function [135–137]. Water treatment processes often involve the use of chlorine and other disinfectants to eliminate microbial contaminants. However, the reaction between chlorine and organic matter in water can lead to the formation of disinfection by-products (DBPs), such as trihalomethanes (THMs) and haloacetic acids (HAAs) [138–140]. Long-term exposure to elevated levels of DBPs in drinking water has been associated with an increased risk of cardiovascular diseases, particularly in relation to heart disease and adverse cardiac remodeling [138–140]. Microplastics are tiny plastic particles that have become a pervasive environmental pollutant, including in water sources. While the direct impact of microplastics on cardiovascular health is still being studied, emerging evidence suggests that microplastic exposure may contribute to oxidative stress, inflammation, and endothelial dysfunction, all of which can increase the risk of cardiovascular diseases [141–147]. The specific health effects of water pollution may vary depending on the concentration and duration of exposure. To safeguard cardiovascular health, ensuring access to clean and uncontaminated water sources is crucial, along with implementing effective water treatment and pollution control measures.

The pollutants mentioned, ranging from PM2.5 and cigarette smoke to microplastics, can exacerbate biological mechanisms of aging through various interconnected pathways. Firstly, these pollutants contribute to increased oxidative stress within cells of the cardiovascular system and other organs [148–158]. They generate excessive reactive oxygen species (ROS), overwhelming the body’s antioxidant defense mechanisms and leading to oxidative damage to cellular structures, including DNA, proteins, and lipids. This oxidative stress contributes to the acceleration of aging processes, as well as the development of age-related diseases.

Secondly, exposure to these pollutants can induce increased DNA damage and cellular senescence [159–163]. DNA damage can occur due to direct interaction with the genetic material or through the generation of ROS. Persistent exposure to pollutants can lead to accumulation of DNA damage, impairing the cell’s ability to repair and maintain genomic integrity. In turn, this initiates cellular senescence, a state of irreversible growth arrest characterized by significant phenotypic alterations, including the emergence of the pro-inflammatory senescence-associated secretory phenotype (SASP). This senescence-driven inflammatory milieu contributes to tissue dysfunction and accelerates the aging process.

Furthermore, pollutants have been shown to impact stem cell function [164–174]. Pesticides, tobacco smoke, and heavy metals have been identified as disruptors of stem cell homeostasis, leading to impaired regenerative capacity and tissue repair. This disruption can further contribute to accelerated aging and compromised organ function.

Inflammation is another critical mechanism affected by these pollutants [10, 155, 175–186]. Chronic exposure to pollutants can trigger a sustained inflammatory response within the cardiovascular system and other organs as well. Inflammatory molecules are released, leading to the activation of immune cells and the production of pro-inflammatory mediators. This chronic state of inflammation contributes to tissue damage, promotes aging-related pathologies, and increases the risk of age-related diseases.

Additionally, pollutants can disrupt mitochondrial function, leading to mitochondrial dysfunction [24, 26, 27, 30]. These toxic substances interfere with mitochondrial processes, such as energy production and oxidative phosphorylation. As a consequence, mitochondrial dysfunction occurs, leading to decreased energy availability, increased oxidative stress, and compromised cellular function.

In summary, environmental pollutants exacerbate biological mechanisms of aging through increased oxidative stress, increased DNA damage and cellular senescence, stem cell dysfunction, inflammation, mitochondrial dysfunction, and other interconnected pathways. Pollutants can also alter epigenetic mechanisms of aging [187]. Understanding these mechanisms is crucial for developing strategies to mitigate the detrimental effects of pollutants and promote healthy aging.

### Environmental drivers of unhealthy cerebrovascular and brain aging

Alzheimer’s disease and Parkinson’s disease are the two most prevalent age-related neurodegenerative diseases affecting the central nervous system. These conditions have a profound impact on cognitive function, motor control, and overall quality of life, particularly in the elderly population. As individuals age, the incidence of both Alzheimer’s and Parkinson’s diseases increases, highlighting the significant burden these conditions pose on global health [188–190]. The pathogenesis of Alzheimer’s and Parkinson’s diseases involves a complex interplay of genetic, environmental, and lifestyle factors. While the exact causes of these diseases remain to be fully elucidated, several mechanisms have been proposed to contribute to their development and progression. In the case of Alzheimer’s disease, important hallmarks are microvascular pathologies (amyloid angiopathy, microhemorrhages, blood–brain barrier disruption) [191–201] and accumulation of amyloid-beta plaques and tau tangles in the brain. These abnormal protein aggregates disrupt normal neuronal communication and function, leading to cognitive decline. Importantly, hypertension has been found to exacerbate several manifestations of Alzheimer’s disease [55, 202–205]. Aging itself plays a crucial role in the development of Alzheimer’s disease by contributing to increased oxidative stress [148, 206, 207], impaired mitochondrial function [25], DNA damage [208–211], and inflammation [212]. These aging-related processes can have significant implications for the cerebral microcirculation, leading to the emergence of microvascular pathologies and promoting the formation and accumulation of amyloid-beta and tau pathology, the hallmark features of Alzheimer’s disease. Additionally, genetic factors, such as mutations in genes like amyloid precursor protein (APP) and presenilin 1 and 2 (PSEN1 and PSEN2), can further increase the risk of developing Alzheimer’s disease.

Parkinson’s disease, on the other hand, is characterized by the degeneration of dopaminergic neurons in the substantia nigra region of the brain. This neuronal loss leads to motor symptoms such as tremors, rigidity, and bradykinesia. Aging is a significant risk factor for Parkinson’s disease, as the brain undergoes age-related changes that contribute to the vulnerability of dopaminergic neurons. These changes include mitochondrial dysfunction, impaired protein handling and clearance mechanisms, oxidative stress, and inflammation. Additionally, genetic factors, such as mutations in the alpha-synuclein (SNCA) gene and genes involved in mitochondrial function, can increase the susceptibility to Parkinson’s disease.

Cognitive impairment caused by macrovascular (atherosclerosis) and microvascular pathologies (vascular cognitive impairment or VCI) is the second most common form of age-related cognitive decline [213–217]. Microvascular pathologies also play a central role in the pathogenesis of Alzheimer’s disease [55, 191, 193, 194, 198, 218, 219]. Age-related changes in the microvasculature [220–225], including alterations in endothelial function [59, 226, 227], blood–brain barrier integrity [199, 200, 228], and cerebral blood flow regulation, contribute to cognitive impairment and the development of neurodegenerative diseases.

Environmental toxicants can adversely affect cerebrovascular and brain health. Common sources of environmental pollutants linked to neurotoxic manifestations include pesticides, solvents, industrial waste, automobile exhaust, cigarette smoke and burning of terrestrial waste. Growing epidemiological and experimental evidence suggests that exposure to environmental toxicants, such as pesticides [229], heavy metals (e.g., lead) and organic solvents (e.g., trichloroethylene, n-hexane, and others) [230, 231], exerts neurotoxic effects and may increase the risk of developing Alzheimer’s and Parkinson’s diseases [25]. These toxicants may damage the cerebral microvasculature and also can cross the blood–brain barrier, leading to cytotoxic effects, neuroinflammation, and consequential neuronal dysfunction and injury. Ambient outdoor air pollution has also been implicated in the exacerbation of the pathogenesis of both Parkinson’s and Alzheimer’s diseases [148, 208, 232–242]. Longitudinal cohort studies have shown associations between increased levels of PM2.5 (fine particulate matter) and a higher hazard of hospital admission for Parkinson’s disease and Alzheimer’s disease and related dementias [243]. Neurovascular damage and cerebromicrovascular dysfunction are increasingly recognized as important contributors to cognitive decline and neurodegeneration [59, 199–201, 244–246]. Cells of the neurovascular unit, including cerebromicrovascular endothelial cells, pericytes, astrocytes, and perivascular microglia, are sensitive to the harmful effects of environmental toxicants [29, 247, 248].

The cellular and molecular mechanisms underlying the impact of environmental toxicants on neurodegeneration and neurovascular injury can involve mitochondrial dysfunction [25–31], which impairs cellular energy production, metabolism, alters intracellular signaling, promotes increased free radical production, and apoptosis. Environmental toxicants can also exacerbate oxidative stress [208], which is causally linked to microglia activation and neuroinflammation [10], cellular senescence [249, 250], and protein aggregation, ultimately leading to neuronal damage and death. It is likely that air pollution contributes to neuronal injury, oxidative stress, neuroinflammation, and cerebromicrovascular impairment, thereby exacerbating the pathogenesis of neurodegenerative diseases [148, 208, 235, 237, 241, 242]. The complex interplay between environmental toxicants and the underlying cellular and molecular mechanisms highlights the importance of understanding and mitigating the impact of these environmental drivers to promote brain health and reduce the burden of cerebrovascular and neurodegenerative disorders.

### Pulmonary diseases

Aging has a significant impact on the incidence and development of pulmonary diseases, particularly chronic obstructive pulmonary disease (COPD [251–254]) and other respiratory disorders. In 2019, there were 212.3 million prevalent cases of COPD reported globally, resulting in 3.3 million deaths and accounting for 74.4 million DALYs (disability-adjusted life years) [255]. The prevalence and death rate of COPD show an increasing tendency with age, peaking in the oldest age group (≥ 95 years) [255]. Studies have revealed that the prevalence of COPD is two to three times higher in individuals over the age of 60 years compared to younger age groups [256]. Furthermore, there are striking similarities between the mechanisms of lung aging and COPD, including cell senescence, shortened telomeres, inflammation, and oxidative stress, suggesting that accelerated aging processes may be involved in the pathogenesis of COPD [256–259].

Ambient particulate matter, a major component of outdoor air pollution, is considered a significant risk factor for respiratory disorders such as COPD. While tobacco smoking was traditionally seen as the primary cause of COPD, it is now recognized that air pollution, including fine particulate matter, plays a substantial role in the development and progression of the disease. According to the World Health Organization (WHO), 18% of premature deaths related to outdoor air pollution are attributed to COPD, making it the most prevalent chronic respiratory disorder [260]. Additionally, 25% of deaths from chronic COPD can be attributed to exposure to household air pollution, primarily in low- and middle-income countries. Certain occupational environments, such as coal and hard-rock mining, construction work, and various manufacturing industries (e.g., concrete, plastics, textiles, rubber, leather, and food products), pose a high risk for COPD [261–263].

The cellular and molecular mechanisms underlying the impact of air pollution and other environmental toxicants on the genesis of pulmonary diseases involve several interconnected pathways. These include increased oxidative stress, DNA damage, cellular senescence, inflammation, and mitochondrial dysfunction. Exposure to air pollution and environmental toxicants leads to an imbalance between the production and neutralization of ROS, resulting in increased oxidative stress. This oxidative stress contributes to cellular injury, DNA damage and induction of senescence, inflammation, and the activation of signaling pathways involved in the pathogenesis of pulmonary diseases. Chronic inflammation, triggered by environmental toxicants, can perpetuate tissue damage and contribute to the progression of pulmonary diseases. Additionally, mitochondrial dysfunction, caused by exposure to pollutants, disrupts cellular energy production and metabolism, further compromising lung function.

### Malignant diseases

The etiology of cancer is multifaceted, influenced by a wide range of factors, and varies depending on the specific type of cancer. Many cancers are considered quintessential diseases of aging (e.g., colorectal cancer, multiple myeloma [264]), as their incidence increases exponentially with age, and mechanisms associated with aging contribute to their pathogenesis [265, 266]. In animal models, interventions and genetic manipulations that delay aging and extend lifespan, such as caloric restriction, have been shown to exert significant anti-cancer effects [267–274]. Conversely, interventions and genetic manipulations that accelerate aging and shorten lifespan promote tumorigenesis and cancer progression [275–277].

Numerous environmental factors have been causally linked to the genesis of various cancer types. Outdoor air pollution, for example, has been identified as a cause of lung cancer occurrence [278, 279] and is increasingly being associated with other types of cancer, including bladder cancer and breast cancer [280]. Indoor air pollution resulting from the burning of solid fuels has been associated with oral, cervical, and esophageal cancer [279].

Workplace or home exposure to a wide range of chemicals has also been causally linked to the development of diverse types of cancer. Asbestos, silica, diesel exhaust, uranium, arsenic, beryllium, cadmium, silica, vinyl chloride, nickel compounds, chromium compounds, coal products, mustard gas, and chloromethyl ethers are significant risk factors for lung cancer [281–291]. Exposure to vinyl chloride increases the risk of liver cancer [292–295], while limited evidence suggests an increased risk with exposure to arsenic [296] and trichloroethylene [297, 298].

The International Agency for Research on Cancer (IARC) classifies asbestos as a cause of ovarian cancer, as well as other cancers [299]. Long-term workplace exposure to polycyclic aromatic hydrocarbons (PAH) and already banned chemicals like arylamines is linked to bladder cancer [300, 301]. Chemicals used in the rubber production industry, coal and tin mining and metal processing increase the risk of gastric cancer, and exposure to asbestos and inorganic lead compounds has limited evidence linking them to gastric cancer [302–304]. Occupational exposure to trichloroethylene, organochlorine and organophosphate pesticides increases the risk of non-Hodgkin’s lymphoma [305–308]. Importantly, women’s hairdresser and textile occupations increase non-Hodgkin’s lymphoma risk [308].

Laryngeal cancer risk is elevated with exposure to coal dust, paint fumes, diesel fumes, formaldehyde, nickel, isopropyl alcohol, and asbestos [309–316]. Furthermore, there are other environmental toxicants known to cause cancer that should be considered. Glyphosate, a commonly used herbicide, has raised concerns and is being investigated for its potential carcinogenic effects [317–321]. Benzene, a chemical found in gasoline, industrial solvents, and tobacco smoke, is a known carcinogen associated with various cancers, including leukemia and multiple myeloma [322–329]. Ionizing radiation, such as in-house exposure to radon, is a risk factor for lung cancer and possibly ovarian cancer [330]. Exposure to ultraviolet (UV) radiation is a major risk factor for most melanomas [331].

The cellular and molecular mechanisms of aging that are exacerbated by the aforementioned environmental factors and exposures contribute to tumorigenesis. These mechanisms include increased production of ROS, DNA damage, genetic instability, and various other pathways associated with aging. The interplay between these aging mechanisms and the effects of environmental toxicants contributes to the initiation and progression of malignant diseases. Importantly, there is evidence that the inflammatory milieu maintained by senescent cells contribute to the development of metastases [332–336]. Understanding the impact of environmental toxicants on accelerated aging and cancer development is crucial for implementing preventive measures, promoting environmental regulations, and reducing the burden of cancer in aging populations. Continued research and vigilance are needed to identify and mitigate the risks associated with exposure to environmental toxicants and their role in cancer incidence.

### Diseases of the musculoskeletal system

Aging of the musculoskeletal system plays a significant role in the pathogenesis of common diseases that have a negative impact on the quality of life, such as osteoporosis, sarcopenia, rheumatoid arthritis, and osteoarthritis [337]. Osteoporosis, characterized by a dysregulation of osteoclast and osteoblast function, is influenced by age-related mechanisms including the accumulation of senescent cells, heightened inflammation, mitochondrial dysfunction, and dysregulated autophagy [338–341]. Mechanisms underlying the pathogenesis of osteoporosis involve endocrine changes, dysfunction of myo-satellite cells, increased inflammation, elevated reactive oxygen species production, macromolecular damage, and dysregulation of proteostasis, cellular energetics, and mitochondrial function [342–357]. Sarcopenia, the gradual decline in muscle mass and strength, contributes to frailty and increases the risk of falls and life-threatening bone fractures in older adults, particularly when combined with osteoporosis. The mechanisms underlying sarcopenia involve impaired muscle protein synthesis, dysregulation of anabolic and catabolic signaling pathways, mitochondrial dysfunction, increased oxidative stress, and altered muscle stem cell function, all contributing to the progressive loss of muscle mass and strength with advancing age. Rheumatoid arthritis, a chronic autoimmune disease affecting the joints, also exhibits an increased incidence with age. The aging of the immune system, known as immunosenescence, and subsequent dysregulation of inflammatory processes are implicated in the pathogenesis of rheumatoid arthritis [358].

There are several environmental toxicants that have been linked to the development of musculoskeletal diseases.

Accumulating evidence suggests that both outdoor and indoor air pollution have detrimental effects on musculoskeletal aging. Two comprehensive studies, involving over 9 million individuals aged 65 years and older over an 8-year period, have demonstrated an association between poor air quality and longitudinal bone loss. Individuals living in areas with higher concentrations of PM2.5 particles were found to have a greater risk of osteoporotic fractures [359]. Furthermore, emerging evidence suggests a potential link between ambient air pollution and arthritis [360, 361]. Household air pollution exposure may also play a significant role in the development of arthritis, particularly in low- and middle-income countries [362]. Understanding the effects of air pollution on musculoskeletal aging is of great importance for public health. Mitigating exposure to poor air quality and implementing measures to improve air pollution levels may help prevent or reduce the burden of musculoskeletal diseases associated with aging. Further research is needed to elucidate the underlying mechanisms linking air pollution and musculoskeletal disorders, as well as to explore potential interventions and strategies for promoting healthy musculoskeletal aging in an increasingly polluted environment.

Exposure to lead, commonly found in old paint, contaminated water, and certain occupational settings, has been associated with various musculoskeletal disorders [363–365]. Lead exposure can impair bone health, leading to decreased bone mineral density, increased fracture risk, and disturbances in bone remodeling. Certain organophosphate pesticides used in agricultural practices have been implicated in musculoskeletal disorders [366]. Prolonged exposure to these pesticides has been associated with decreased grip strength, muscle weakness, and altered neuromuscular function. Cadmium is a toxic metal present in certain industrial processes, batteries, and cigarette smoke. Prolonged exposure to cadmium has been linked to adverse effects on bone health, including decreased bone mineral density, osteoporosis, and an increased risk of fractures [367]. Polychlorinated biphenyls (PCBs) are persistent organic pollutants that were widely used in electrical equipment and industrial applications. Exposure to PCBs has been associated with adverse effects on the musculoskeletal system [368–370].

Certain toxicants have been associated with an increased risk or exacerbation of arthritis. For example, occupational exposure to crystalline silica, commonly found in industries such as mining, construction, and manufacturing, has been linked to an increased risk of developing rheumatoid arthritis [371–375]. It is also possible that prolonged exposure to benzene, a chemical commonly found in industrial settings and certain products such as gasoline, and to vinyl chloride, commonly found in the plastics industry, may also increase the developing of autoimmune diseases, such as arthritis [376, 377].

The mechanisms by which environmental toxicants exacerbate musculoskeletal diseases are complex and involve multiple pathways, including impaired satellite cell function, dysregulation of hormonal signaling, mitochondrial dysfunction, disruption of intracellular signaling pathways involved in muscle protein turnover induction of oxidative stress and inflammation.

## Perspectives

In conclusion, the impact of environmental factors on unhealthy cardiovascular, cerebrovascular, and brain aging cannot be overstated. The integration of geroscience, environmental health sciences, and toxicology is crucial in bridging gaps and enhancing our understanding of the relationship between environmental drivers and aging processes. This multidisciplinary approach promotes collaborative efforts and enables a comprehensive assessment of the complex interplay between environmental factors and aging outcomes.

The exposomic framework has emerged as a powerful tool in advancing our understanding of the associations between environmental factors and healthy aging. By capturing the totality of environmental exposures throughout an individual’s life, the exposomic approach provides a holistic perspective on the cumulative effects of these exposures on aging processes. While capturing exposures over a lifetime poses challenges and requires resources, leveraging historical data sources such as retrospective surveys or longitudinal cohorts offers a practical avenue for conducting research in this area. By harnessing existing data, researchers can gain valuable insights into the long-term impact of environmental exposures on aging and identify potential strategies for promoting healthy aging in the population.

Biomarkers of biological age have emerged as valuable tools for assessing the effects of environmental toxicants on aging. These biomarkers provide objective measures of an individual’s physiological state and reflect the cumulative impact of genetic, lifestyle, and environmental factors on the aging process. Telomere length, epigenetic modifications, DNA damage markers, inflammation markers, oxidative stress markers, and mitochondrial function indicators are among the commonly used biomarkers of biological age. Assessing the effects of environmental toxicants on aging through biomarkers allows for a comprehensive understanding of the underlying mechanisms and helps identify individuals at higher risk of accelerated aging or age-related diseases.

Emerging methodologies for determining biological age, such as epigenetic clocks, proteomic clocks, and lipidomic clocks, have brought new insights to the field of aging research [378–383]. Epigenetic clocks utilize DNA methylation patterns to predict biological age, while proteomic clocks assess changes in protein levels and modifications associated with aging. By integrating these clocks with physiological measurements, researchers can obtain a more comprehensive understanding of an individual’s biological age and the factors influencing the rate of aging. These methodologies hold great potential for advancing our understanding of healthy aging, identifying individuals at higher risk of age-related diseases, and developing targeted interventions to promote healthier and more vibrant aging.

Integrating the analysis of biological age and biomarkers of aging with epidemiological and toxicological studies allows for a more comprehensive assessment of the relationship between environmental toxicants and aging. This multidimensional approach provides valuable data for developing strategies to mitigate the adverse effects of toxicants, promoting healthier aging, and informing public health policies aimed at reducing exposure to harmful environmental factors. Longitudinal studies tracking changes in biomarker profiles and the subjects’ biological age in response to environmental exposures can provide valuable information on the impact of toxicants over time. Additionally, biomarker assessments can aid in evaluating the effectiveness of interventions or preventive measures aimed at mitigating the detrimental effects of toxicants on aging.

A geroscience approach to environmental health sciences will further identify potential new areas of interdisciplinary research. Environmental toxicants can exacerbate mitochondrial DNA damage and mutagenesis, accelerating mitochondrial aging and promoting dysfunction, cellular energetic dysfunction, apoptosis signaling, and increased production of mitochondria-derived free radicals [384]. Future epidemiological studies should aim to characterize in detail the impacts of environmental chemical exposures on mitochondrial DNA mutagenesis, linking it to the genesis of accelerated aging phenotypes and the incidence of age-related non-communicable diseases.

Furthermore, intersecting area-level indicators with trends in biological aging and the incidence of age-related diseases in a population opens new horizons in epidemiology. By employing this approach, longitudinal studies can lead to a deeper understanding of the role of the exposome in modulating biological aging processes and its contribution to health inequalities in aging. Identifying the role of environmental toxicants in accelerated aging and the increased prevalence of cognitive impairment, dementia, and other age-related diseases in vulnerable populations will advance opportunities for intervention and prevention.

In summary, by advancing our knowledge of the environmental drivers of unhealthy aging and employing an integrative approach, we can develop effective strategies to promote healthier aging, mitigate the detrimental effects of environmental toxicants, and improve the overall well-being of the aging population.
